# Decrease of the DNA methylation levels of the *ADRB3* gene in leukocytes is related with serum folate in eutrophic adults

**DOI:** 10.1186/s12967-018-1529-0

**Published:** 2018-06-05

**Authors:** Yohanna de Oliveira, Raquel Patrícia Ataíde Lima, Rafaella Cristhine Pordeus Luna, Mussara Gomes Cavalcanti Alves Monteiro, Cássia Surama Oliveira da Silva, Rayner Anderson Ferreira do Nascimento, Keylha Querino de Farias Lima, Ana Hermínia Andrade e Silva, Flávia Emília Leite de Lima Ferreira, Rodrigo Pinheiro de Toledo Vianna, Ronei Marcos de Moraes, Naila Francis Paulo de Oliveira, Aléssio Tony Cavalcanti de Almeida, Alexandre Sérgio Silva, Alcides da Silva Diniz, Maria José de Carvalho Costa, Maria da Conceição Rodrigues Gonçalves

**Affiliations:** 1 Health Sciences Center (Centro de Ciências da Saúde), Departament of Nutrition, Federal University of Paraíba (Universidade Federal da Paraíba), João Pessoa, Brazil; 2 Center of Exact Sciences and Nature (Centro de Ciências Exatas e da Natureza), Federal University of Paraíba (Universidade Federal da Paraíba), João Pessoa, Brazil; 3 Departament of Statistics, Center of Exact Sciences and Nature (Centro de Ciências Exatas e da Natureza), Federal University of Paraíba (Universidade Federal da Paraíba), João Pessoa, Brazil; 4 Departament of Molecular Biology, Center of Exact Sciences and Nature (Centro de Ciências Exatas e da Natureza), Federal University of Paraíba (Universidade Federal da Paraíba), João Pessoa, Brazil; 5 Department of Economics, Center for Applied Social Sciences (Centro de Ciências Sociais Aplicadas), Federal University of Paraíba (Universidade Federal da Paraíba), João Pessoa, Brazil; 6 Departament of Nutrition, Health Sciences Center (Centro de Ciências da Saúde), Federal University of Pernambuco (Universidade Federal de Pernambuco), Recife, Brazil

**Keywords:** *ADRB3*, DNA methylation, Body mass index, Folic acid

## Abstract

**Background:**

DNA methylation has been evidenced as a potential epigenetic mechanism related to various candidate genes to development of obesity. Therefore, the objective of this study was to evaluate the DNA methylation levels of the *ADRB3* gene by body mass index (BMI) in a representative adult population, besides characterizing this population as to the lipid profile, oxidative stress and food intake.

**Methods:**

This was a cross-sectional population-based study, involving 262 adults aged 20–59 years, of both genders, representative of the East and West regions of the municipality of João Pessoa, Paraíba state, Brazil, in that were evaluated lifestyle variables and performed nutritional, biochemical evaluation and DNA methylation levels of the *ADRB3* gene using high resolution melting method. The relationship between the study variables was performed using analyses of variance and multiple regression models. All results were obtained using the *software* R, 3.3.2.

**Results:**

From the stratification of categories BMI, was observed a difference in the average variables values of age, waist-to-height ratio, waist-to-hip ratio, waist circumference, triglycerides and intake of trans fat, which occurred more frequently between the categories “eutrophic” and “obesity”. From the multiple regression analysis in the group of eutrophic adults, it was observed a negative relationship between methylation levels of the *ADRB3* gene with serum levels of folic acid. However, no significant relation was observed among lipid profile, oxidative stress and food intake in individuals distributed in the three categories of BMI.

**Conclusions:**

A negative relationship was demonstrated between methylation levels of the *ADRB3* gene in eutrophic adults individuals with serum levels of folic acid, as well as with the independent gender of BMI, however, was not observed relation with lipid profile, oxidative stress and variables of food intake. Regarding the absence of relationship with methylation levels of the *ADRB3* gene in the categories of overweight, mild and moderate obesity, the answer probably lies in the insufficient amount of body fat to initiate inflammatory processes and oxidative stress with a direct impact on methylation levels, what is differently is found most of the times in exacerbated levels in severe obesity.

**Electronic supplementary material:**

The online version of this article (10.1186/s12967-018-1529-0) contains supplementary material, which is available to authorized users.

## Background

Obesity is considered a serious public health problem, affecting 39% of the world population [1]. The epidemic projection is that by 2025, more than 2 billion adults are overweight or obese [2].

Multiple biological, behavioural, genetic and epigenetic determinants are contributing to the multifactorial aetiology of obesity, for example, sleep disturbances, potential traits of obesogenic behavior, characterized by excess calorie intake and sedentary lifestyle, peripheral and central regulation of energy balance, adipose tissue and skeletal muscle biology, in addition to changes from the gut microbiota, hormone signalling, reproductive factors, drugs, and intrauterine and epigenetic intergenerational effects [3].

Increasing evidence indicates that epigenetic modifications in adipose tissue may be involved in pathogenesis of metabolic disorders, fat storage, cell remodeling and adipogenesis, emphasizing the involvement of DNA methylation in differentiation of adipocytes and its effect on expression of genes related to obesity [4].

DNA methylation is the most frequent and well-characterized epigenetic mark, for playing a key role on regulating gene expression and at molecular phenotype, without any change in the sequence of DNA, it can be altered by environmental expositions and genetic influences [5]. In addition, folate is a water-soluble B vitamin which plays an important role as donor of methyl groups required for proper epigenetic regulation [6].

Another complexity encountered in studying obesity is the marked heterogeneity of obese individuals, through the notable differences in body configuration and in the regional accumulation of body fat in adiposity levels, such as subcutaneous obesity, in which excess subcutaneous fat is found around the hip and thigh areas, and visceral obesity, in which fat is mainly concentrated in the abdominal region, mainly mesenteric adipose tissue. Visceral obesity, also known as the excess of visceral adipose tissue (VAT), tends to be more pernicious in health terms, being a potential factor for cardiovascular risk [7].

Recently, it was demonstrated the modification in methylation levels of candidate genes to development of obesity exhibited a relation excellent in the discrimination of obesity from non-obesity status, thus suggesting that DNA methylation levels in blood leukocytes has great potential for to characterize the obesity phenotype by to identify robust and biologically relevant epigenetic variation related to body mass index (BMI), using an easily accessible and minimally invasive biological material instead of adipose tissue [8].

Interestingly, the beta-3 adrenergic receptor (*ADRB3*) is located on chromosome 8, which contains two exons and 2.252 base pairs, and is known to play a key role in the regulation of lipolysis in white and brown adipose tissues, which provides free fatty acids for thermogenesis, indicating the participation of the *ADRB3* gene in the regulation of body weight in humans [9]. However, the role of the DNA methylation of the *ADRB3* gene related to the increase of susceptibility to visceral obesity and alter the body fat distribution have not been fully elucidated yet.

In this context, the present study aims to evaluate the methylation levels of the *ADRB3* gene by BMI in a representative adult population, in addition to characterizing this population as to the lipid profile, oxidative stress and food intake.

## Methods

### Study design

This is a cross-sectional epidemiological study linked to the project titled: “II Cycle of Diagnosis and Intervention of Food and Nutritional Status and the Most Prevalent Non-Communicable Diseases in the Population of the Municipality of João Pessoa/PB” (II Ciclo de Diagnóstico e Intervenção da Situação Alimentar, Nutricional e das Doenças não Transmissíveis mais Prevalentes da População do Município de João Pessoa/PB, II DISANDNT/PB), which was conducted between May 2015 and May 2016.

### Ethical issues

The research protocol of the project mentioned above, to which the present study is linked, was submitted and approved by the Research Ethics Committee of the Health Sciences Center (Centro de Ciências da Saúde, CCS) of the Federal University of Paraíba (Universidade Federal da Paraíba, UFPB), under the Protocol Number 0559/2013, in accordance with the ethical standards for research involving human beings included in Resolution 466 of the December 12, 2012, meeting of the National Health Council/National Research Ethics Committee.

After identifying the residences in randomly selected city blocks located in the East and West areas of the city of João Pessoa, the researchers introduced themselves to the residents, explaining the purpose of the study and requesting their participation. To respect ethical guidelines for research involving humans, the participants residing in the selected households were included in the study only when they had consented to participation by signing the Informed Consent form.

### Sampling

To conduct this population-based study, representative of the East and West regions of the municipality of João Pessoa, a representative sample of the adult group was calculated using information provided by the city hall, such as the city map, number of blocks per neighborhood, and data from the Brazilian Institute of Geography and Statistics (Instituto Brasileiro de Geografia e Estatística, IBGE) [10]. In the present study, two regions (East and West) with similar socioeconomic, epidemiological and lifestyle characteristics in relation to the other regions were selected in order to represent the four regions of the municipality.

For the sample calculation, a single multi-level sampling procedure was used. Due to the presence of heterogeneity in income and the relationship between income, disease prevalence and nutrition [11], stratified sampling was used [12] with the city blocks at the first level. In this level, the neighborhoods/city blocks of the East and West zones of the municipality were ranked by income class into four strata according to information obtained from the IBGE [10].

After stratification, the sample size, or number of representative city blocks per zone, was calculated. Next, the weight of each stratum was calculated as the number of city blocks per zone according by strata, according to the formula defined by Silva, Moraes and Costa [13].

Considering that the average income of adults in the East and West areas was R$2213.26 [data obtained from “The first cycle of diagnoses and intervention of the food and nutritional situation, and of the most prevalent communicable diseases of the municipal population of João Pessoa/PB” (07/2008-01/2010)], with a standard deviation of R$2601.93 and a margin of error of R$3320.00 in income, the minimum sample of adults in João Pessoa necessary to be statistically representative of the eastern and western areas (with a confidence level of 95%) was 236 adults. Thus, a sample of 262 adults distributed across the study area was selected.

This study had the following inclusion criteria: individuals aged 20–59 years; different socioeconomic conditions, and medication users and non-users, with the exception of the drugs described below. Exclusion criteria included the following: individuals with neuropsychiatric disorders; multivitamin, mineral, anorexigen, and anabolic supplement users; and pregnant and lactating women.

### Data collection

The household visits and application of the questionnaires were conducted by graduate students of the Nutrition Programs. These are Master’s and doctoral students in the Post Graduate Program in Nutrition Sciences (Programa de Pós Graduação em Ciências da Nutrição, PPGCN) at UFPB who were trained before initiating data collection after the pilot study was conducted. Questionnaires were applied for demographic, socioeconomic, and epidemiological characterization, lifestyle, nutritional and food intake assessment, and also biochemical evaluation.

### Nutritional assessment

Weight and height measurements were taken in triplicate, and the average of the three values was used. The BMI was then calculated using the body weight (kg) divided by the squared body height (meters), and the cut-off points recommended in adults aged 20–59 years old by the World Health Organization (WHO) were used [14].

Waist circumference (WC) was used to determine abdominal obesity, proceeding the American Heart Association (AHA) cut-off points of ≥ 88 cm in women and ≥ 102 cm in men [15]. The waist-hip ratio (WHR) was used to define the abdominal obesity status, defined as a WHR > 0.90 in men and > 0.85 in women [16].

The waist-to-height ratio (WHtR) was based on the quotient of the waist and height measurements in centimeters. The cut-off point used was 0.5, as supported by studies conducted around the world in diverse population as an evaluation tool to identify health risks and related morbidities [17].

### Dietary assessment

To evaluate the regular food intake of the individuals, three 24-h dietary recalls (24HR) were performed, contemplating one at the weekend, with a 15-day interval from the beginning of the data collection, according to a study previously published [18].

### Biochemical analysis

The blood samples were collected from the individuals after a 12-h fasting at home by an experienced nurse, and the analysis of biochemical variables were performed according to a study previously published [18]. The analysis of alpha-1 acid glycoprotein (AGP) was measured using the immunoturbidimetry technique and tests were performed in an automated analyzer (LabMax 240, Labtest, Lagoa Santa, MG, Brazil) using standardized kit following the instructions provided by the manufacturer (Labtest, Lagoa Santa, MG, Brazil).

The Access Folate assay is a paramagnetic particle, chemiluminescent immunoassay for the quantitative determination of folic acid levels in human serum and plasma (heparin) or red blood cells (RBC) using the Access Immunoassay Systems. Folate levels in serum and plasma or RBC are used to assess folate status. The serum folate level is an indicator of recent folate intake, while that a low RBC folate value can indicate a prolonged folate deficiency. Thus, serum levels of folate were estimated using commercial kit (Access Folate Kit [A98032]; Beckman Coulter, Fullerton, CA, USA). Folate levels were determined by a corpuscle immune chemiluminescence assay (Access 2 Immunoassay System; Beckman Coulter, Fullerton, CA, USA). The assay was performed in accordance with the manufacturers’ protocol. Folate deficiency was defined as < 3.10 ng/ml with analytical sensitivity of 0.5 ng/ml.

### Analysis of DNA methylation levels

The leukocyte DNA was isolated, quantified and transformed with sodium bisulfite according to the conditions described in a previously published study [18]. The analysis of methylation levels in genomic DNA from blood was performed by High Resolution Melting (HRM) Real Time PCR method in a applied biosystems 7500 fast system. PCR was performed in a total volume of 20 μl containing: 1× buffer, 4 mM Mg^2+^, 200 μM of each dNTPs (Qiagen), 250 nM of each primer, 5 μM SYTO^®^ (Invitrogen), 1 U HotstarTaq DNA Polymerase (Qiagen) and 1 μl of bisulfite-modified DNA. Primers were designed from the genome sequence deposited in the UCSC genome browser http://genome-euro.ucsc.edu/(chr8:37,962,991-37,966,965), F:5′-TAGGTGATTTGGGAGATTTTTTTT-3′ and R:5′-CCCCTAACAACCCACTAATATTAAC-3′. The PCR program consisted of an initial enzymatic activation at 95 °C for 10 min, followed by 50 cycles of 45 s at 95 °C, 45 s at 60 °C and 45 s at 72 °C and the final extension at 72 °C for 10 min. The melting curves were normalized by calculation of the ‘line of best fit’ in between two normalization regions before and after the major fluorescence decrease representing the melting of the PCR product using the software provided with the HRM Software v2.0, provided by 7500 fast system.

## Statistical analysis

All statistical analyses were performed with R software, 3.3.2 [19]. Initially it was performed the characterization of the sample by descriptive statistics represented by a single frequency, using position measurements, such as central tendency and dispersion. The data were tested for normality using Lilliefors test, which is a derivative of the Kolmogorov–Smirnov test. Moreover, analysis of variance (ANOVA) was used to verify if there was a mean difference between the BMI in which subjects were classified, for all quantitative variables of the study.

To identify the existence of statistically significant relationships between DNA methylation levels of the *ADRB3* gene and variables of this study, the following multiple linear regression models were used:

Model 1:

$$\begin{aligned} {\text{Methylation levels}}\, = & \,\beta 0\, + \,\beta 1\,*\,{\text{Total cholesterol}}\, + \,\beta 2\,*\,{\text{LDL}}\, + \,\beta 3\,*\,{\text{HDL}} \\ & + \,\beta 4\,*\,{\text{Triglycerides}}\, + \,\beta 5\,*\,{\text{Homocysteine}}\, + \,\beta 6\,*\,{\text{TAC}}\, + \,\beta 7\,*\,{\text{MDA}} \\ & + \,\beta 8\,*\,{\text{Alpha-}} 1 {\text{ acid glycoprotein}}\, + \,\beta 9\,*\,{\text{Folic acid}}\, + \,\beta 10\,*\,{\text{Vitamin B12}}. \\ \end{aligned}$$

Model 2:

$$\begin{aligned} {\text{Methylation levels}}\, = & \,\beta 0\, + \,\beta 1\,*\,{\text{Calories}}\, + \,\beta 2\,*\,{\text{Total fat}}\, + \,\beta 3\,*\,{\text{Folate}} \\ & + \,\beta 4\,*\,{\text{Vitamin B12}}\, + \,\beta 5\,*\,{\text{Monounsaturated fat}}\, + \,\beta 6\,*\,{\text{Oleic acid}} \\ & + \,\beta 7\,*\,{\text{Omega 3}}\, + \,\beta 8\,*\,{\text{Omega 6}}\, + \,\beta 9\,*\,{\text{Saturated fat}}\, + \,\beta 10\,*\,{\text{Cholesterol}} \\ & + \,\beta 1 1\,*\,{\text{Polyunsaturated fat}}\, + \,\beta 1 2\,*\,{\text{Trans fat}}. \\ \end{aligned}$$

Considering 5% as significance.

## Results

The sample comprised 262 adults individuals, being 175 female (66.79%) and 87 males (33.21%).

In Table 1, it was performed the multiple comparison testing of means for each variable, through the ANOVA test, it was observed a difference in mean variables values described below, in relation to nutritional status: average age, waist-to-height ratio (WHtR), waist-to-hip ratio (WHR), waist circumference (WC), triglycerides and intake of trans fat. It is relevant to mention that methylation levels did not differ significantly by nutritional status.

**Table 1:** Characteristics of adults population by nutritional status of the municipality of João Pessoa/PB/Brazil

Parameter	Eutrophic (43.51%)	Overweight (36.26%)	Obesity (20.23%)	p-value
Methylation levels (%) Mean ± SD	43.29 ± 19.14	39.32 ± 17.57	42.74 ± 17.29	0.6020
Average age Mean ± SD	35.07 ± 11.83^ab^	41.78 ± 12.52^b^	42.23 ± 11.58^a^	0.0002*
WHtR (cm/cm) Mean ± SD	0.40 ± 0.16^ab^	0.55 ± 0.06^bc^	0.61 ± 0.15^ac^	> 0.0000*
WHR (cm/cm) Mean ± SD	0.79 ± 0.08^ab^	0.87 ± 0.09^b^	0.92 ± 0.09^a^	> 0.0000*
WC (cm) Mean ± SD	74.45 ± 9.45^ab^	89.48 ± 8.67^bc^	101.52 ± 13.90^ac^	> 0.0000*
Total cholesterol (mg/dl) Mean ± SD	175.15 ± 35.63	195.73 ± 45.19	199.40 ± 43.48	0.9460
LDL (mg/dl) Mean ± SD	97.74 ± 47.71	112.03 ± 56.16	112.54 ± 62.30	0.1050
HDL (mg/dl) Mean ± SD	45.84 ± 11.70	41.45 ± 10.46	42.87 ± 10.31	0.2740
Triglycerides (mg/dl) Mean ± SD	120.39 ± 63.78^a^	158.10 ± 93.39	158.33 ± 73.90^a^	0.0241*
TAC (%) Mean ± SD	41 ± 15	41 ± 14	42 ± 15	0.6880
MDA Mean ± SD	2.75 ± 0.99	2.98 ± 0.89	2.93 ± 0.75	0.6330
Alpha-1 acid glycoprotein (mg/dl) Mean ± SD	63.96 ± 20.86	64.66 ± 17.67	68.02 ± 16.45	0.2020
Homocysteine (micromol/l) Mean ± SD	11.42 ± 12.40	10.31 ± 6.43	10.69 ± 6.25	0.3540
Serum folate (ng/ml) Mean ± SD	14.23 ± 5.47	13.67 ± 4.87	14.70 ± 5.74	0.5660
Vitamin B12 (pg/ml) Mean ± SD	277.67 ± 136.74	261.83 ± 113.45	289.23 ± 126.91	0.0936
Total fat (g) Mean ± SD	55.12 ± 29.94	58.85 ± 52.14	58.47 ± 46.68	0.8810
Calories (Kcal) Mean ± SD	1805.16 ± 695.07	1829.67 ± 1012.35	1954.71 ± 1815.64	0.7160
Dietary folate (mcg) Mean ± SD	170.58 ± 174.83	139.58 ± 111.68	126.53 ± 89.34	0.5640
Vitamin B12 (mcg) Mean ± SD	2.10 ± 1.53	1.86 ± 1.59	2.01 ± 1.64	0.1370
Monounsaturated fat (g) Mean ± SD	14.50 ± 8.46	16.43 ± 13.80	15.07 ± 11.68	0.9240
Oleic acid (g) Mean ± SD	9.59 ± 7.33	12.03 ± 12.19	11.09 ± 10.75	0.7780
Omega 3 (g) Mean ± SD	0.64 ± 0.95	0.61 ± 0.63	0.68 ± 0.89	0.4100
Omega 6 (g) Mean ± SD	5.81 ± 4.96	6.81 ± 5.64	8.44 ± 14.17	0.2660
Saturated fat (g) Mean ± SD	17.33 ± 10.69	18.42 ± 17.72	16.85 ± 13.36	0.6750
Cholesterol (mg) Mean ± SD	264.93 ± 264.93	258.18 ± 224.64	226.09 ± 171.47	0.9460
Trans fat (g) Mean ± SD	0.75 ± 1.60^a^	0.61 ± 0.54	0.76 ± 0.91^a^	0.0172*

^a^Significant difference in mean values of the variable between obese and eutrophic individuals (p-value Tukey’s test < 0.05)

^b^Significant difference in mean values of the variable between overweight and eutrophic individuals (p-value Tukey’s test < 0.05)

^c^Significant difference in mean values of the variable between obese and overweight individuals (p-value Tukey’s test < 0.05)

To know which of BMI categories there was a significant difference, Tukey’s test was performed, which occurred more frequently between the categories “eutrophic” and “obesity”.

No significant association was observed among the variables methylation levels of *ADRB3* gene with lipid profile and oxidative stress in nutritional status of eutrophic, overweight or obesity. However, it was observed that methylation levels of *ADRB3* gene were negatively associated with serum levels of folic acid in the eutrophic adults group; when folic acid values increased by 1 ng/ml, methylation levels decreased in mean, 2.27% (p-value = 0.0327 < 0.05), according showed in Table 2. When applied multiple regression based on model 1 including age and gender factors in the three categories of BMI observed that there was only relationship for methylation levels and male gender in eutrophics (Additional file 1). This result means that, on average, eutrophics males have 0.1527 more methylation levels than females (p-value = 0.0070 < 0.05).

**Table 2:** Multiple regression analysis of methylation levels of the *ADRB3* gene with lipid profile and oxidative stress of adult individuals by nutritional status

	Coefficient	CI 95%	Statistics t	p-value
Total cholesterol (mg/dl)				
Eutrophic*	0.23	(− 0.10 ± 0.56)	0.69	0.4999
Overweight	0.06	(− 0.04 ± 0.15)	0.61	0.5442
Obesity	− 0.13	(− 0.24 ± − 0.01)	− 1.06	0.3000
LDL (mg/dl)				
Eutrophic*	− 0.47	(− 0.82 ± − 0.11)	− 1.31	0.2051
Overweight	− 0.02	(− 0.09 ± 0.04)	− 0.38	0.7087
Obesity	0.12	(0.03 ± 0.21)	1.35	0.1910
HDL (mg/dl)				
Eutrophic*	0.41	(− 0.16 ± 0.97)	0.72	0.4808
Overweight	0.27	(− 0.02 ± 0.56)	0.92	0.3639
Obesity	0.06	(− 0.49 ± 0.60)	0.12	0.9160
Triglycerides (mg/dl)				
Eutrophic*	0.08	(− 0.06 ± 0.22)	0.54	0.5941
Overweight	0.02	(− 0.01 ± 0.05)	0.57	0.5742
Obesity	0.02	(− 0.04 ± 0.08)	0.36	0.720
TAC (%)				
Eutrophic*	27.64	(− 8.54 ± 63.83)	0.76	0.4543
Overweight	11.30	(− 8.87 ± 31.48)	0.56	0.5789
Obesity	17.42	(− 7.63 ± 42.47)	0.70	0.4940
MDA				
Eutrophic*	− 4.55	(− 1.37 ± 10.48)	0.77	0.4516
Overweight	− 8.07	(− 12.60 ± − 3.53)	− 1.78	0.0839
Obesity	− 1.54	(− 6.81 ± 3.72)	− 0.29	0.7720
Alpha-1 acid glycoprotein (mg/dl)				
Eutrophic*	0.23	(0.02 ± 0.44)	1.07	0.2964
Overweight	0.00	(− 0.18 ± 0.19)	0.02	0.9880
Obesity	0.08	(− 0.16 ± 0.32)	0.33	0.7420
Homocysteine (micromol/l)				
Eutrophic*	− 1.74	(− 3.50 ± 0.01)	− 0.99	0.3339
Overweight	− 0.45	(− 0.82 ± − 0.08)	− 1.20	0.2368
Obesity	− 0.54	(− 1.02 ± − 0.06)	− 1.12	0.2750
Serum folate (ng/ml)				
Eutrophic*	− 2.27	(− 3.26 ± − 1.28)	− 2.30	0.0327*
Overweight	− 0.79	(− 1.53 ± − 0.05)	− 1.06	0.2956
Obesity	− 0.44	(− 1.13 ± 0.25)	− 0.63	0.5330
Vitamin B_12_ (pg/ml)				
Eutrophic*	6.65	(2.72 ± 10.58)	1.69	0.1067
Overweight	3.67	(1.21 ± 6.14)	1.49	0.1452
Obesity	− 0.32	(− 2.80 ± 2.16)	− 0.13	0.8970

* Eutrophic: after adjustment for sex and age significant relationships remained for methylation levels and male gender

Regarding the results of multiple regression based on model 2 described previously ont food intake, was not observed significative relationship between methylation levels of *ADRB3* gene and food intake in the three categories of BMI (Additional file 1).

## Discussion

In the present study, it was observed that methylation levels of *ADRB3* gene were negatively associated with serum levels of folic acid in adult eutrophic individuals. Noting that as serum levels of folic acid in eutrophic individuals increases, methylation levels decreases. However, no significant relationship was observed among lipid profile, oxidative stress and food intake in individuals distributed in the three categories of BMI.

Although the mechanism responsible have not been elucidated yet, higher serum folic acid concentrations were associated with lower DNA methylation levels of *ADRB3* gene in eutrophic individuals, which may represent a protective effect for obesity. This finding in accordance with the results of a study that demonstrated that higher serum folic acid concentrations is associated with lower levels of DNA methylation in leukocytes in women with normal weight, and with a distinctive epigenetic response in women with obesity, providing novel evidences that the adequate folate metabolism may be affected by obesity, due probably to the volumetric dilution, resulting in a redistribution of the vitamin from circulation into tissue [6].

Considering the difference between the forms and bioavailability of folates, the folate family of compounds include folic acid and its derivatives which include 5- methyltetrahydrofolate (5-MTHF), 5-formyltetrahydrofolate (5-FTHF or folinic acid), 10-formyl-THF, 5,10-methylene-THF and unsubstituted THF. Folic acid, a synthetic oxidized form of folate, is used in supplements and added to food because of its high stability and bioavailability. Folinic acid is a 5-formyl derivative of THF. Unlike the synthetic folate, folinic acid is naturally found in food. It is readily converted to THF without requiring the action of the enzyme dihydrofolate reductase (DHFR). Therefore its function as a vitamin is unaffected by drugs inhibiting this enzyme, such as methotrexate. 5-MTHF is a biologically active form of folate and is the most abundant form found in plasma, representing > 90% of folate and is the predominant active metabolite of ingested folic acid [20].

Given the paucity of research on the different BMI categories relating methylation levels in leukocytes in adults of the same population, this study represents the first effort on searching for differences in methylation levels of the *ADRB3* gene in varying degrees of obesity, which may help to understand of relations with different categories of the BMI variable.

While it is well-known that central to the pathophysiology of obesity is an excess amount of adiposity, on BMI (which does not directly estimate body fat) still prevails as the most used indicator of adiposity worldwide, for practical reasons, comparability and ease to measure weight and height [7]. To refine risk assessment, BMI could be complemented by also measuring WC to discriminate between subcutaneous obesity and visceral obesity, the ratio of WHR circumferences and the WHtR, this latter being a clinical indicator supporting of health risk, revealing that the accumulation of VAT and abdominal subcutaneous fat is strongly associated with cardiometabolic risk [21].

Obesity is a heterogeneous disease with many different subtypes and DNA methylation might contribute to these differences [22]. Although few researchers have investigated the relationship of the *ADRB3* gene and its role in susceptibility to obesity, only three studies on methylation were found in the literature consulted, being the first performed with methylation of *ADRB3* gene in severely obese men [23], the second with methylation in VAT in obese and slim individuals [9], and the third with methylation in overweight and obese adult women [18].

In this context, Guay et al. [23] demonstrated that higher DNA methylation levels of *ADRB3* gene in blood were significantly associated with a lower WHR and lower LDL cholesterol levels in severely obese men. Besides that, *ADRB3* g.-843C>T and p.W64R polymorphisms were found to be strongly associated with *ADRB3* DNA methylation, reinforcing the impact of both, mutations as well as DNA methylation at the *ADRB3* gene promoter.

It has also been postulated that the Trp64Arg *ADRB3* genetic variant is associated with 3.9 increase in BMI, and these associations were observed mainly in East Asian populations. In contrast, lower gene expression of the *ADRB3* gene in Caucasians and its relationship with higher propensity to obesity and related comorbidities are still controversial [24].

It was reported that the decrease in expression of *ADRB3* gene in VAT is associated with obesity, but not with the hypermethylation, demonstrating that the average levels of methylation of this gene in VAT was similar in slim individuals and with obesity from grade III [9], corroborating with the present study regarding the absence of difference of the methylation levels of the *ADRB3* gene in leukocytes for different categories of BMI. Thus, the identification in blood such as biomarker easily accessible of the DNA methylation instead of adipose tissue is a promising alternative in the epigenetics field, but still challenging.

However, these findings do not apply to the present study, un precedented regarding methylation levels of the *ADRB3* gene in leukocytes in a representative adult population of both genders by BMI categories. As it does not exist in literature searching studies classifying the population based in the three categories of BMI and methylation levels of the *ADRB3* gene for comparative purposes, it is suggested that probably other factors have influenced the results such as sample size and non-stratification by gender. It is also important to mention that different blood cell populations may present different methylation profiles, as already shown for genes implicated in immune-related disorders [25], however we do not know if this is applicable to the *ADRB3* gene.

Nevertheless, when the gender was analyzed isolated in relation to the methylation profile of *ADRB3* gene, it was observed that males showed higher methylation levels than female (46.40 ± 18.81 and 39.42 ± 17.54 respectively, p = 0.0037; Mann–Whitney). On the other hand, when it evaluated the difference between levels of methylation by age groups no significant results were found (20–39 years old: 42.04 ± 19.12, 40–59 years old: 41.40 ± 17.27; p = 0.9310; Mann–Whitney); (20–30 years old: 39.95 ± 18.63, 31–40 years old: 45.70 ± 19.60, 41–50 years old: 44.39 ± 17.28, 51–59 years old: 39.10 ± 17.04; p = 0.1338; Kruskal–Wallis). Indeed, the literature shows that in spite of external factors such as diet [18], smoking [26], alcohol consumption [27] and air pollution [28], inner factors such as age and gender have also influence on the DNA methylation profile. Differences in the profile of DNA methylation between males and female have already been observed in skin for *miR*-*137* gene [29] and in buccal cells for *MMP9* and *TIMP*-*1* genes [30]. It is noteworthy that sex hormones may regulate DNA methylation differentially in the context of different tissues, developmental stages, and pathological conditions [31].

On the other hand, it is relevant to mention that it was not detectable a relation between the methylation levels of the *ADRB3* gene with overweight and obesity, since some studies have shown that these relations are more prominent in severe obesity (grade III e IV) [9, 23]. Suggesting that, in overweight, mild and moderate obesity (grade I e II), the state in which starts triggers the inflammatory cascade and the impacts of oxidative stress, the amount of adiposity is still insufficient to influence the epigenetic marks and result in alterations in phenotype.

Interestingly, DNA methylation, especially in promoter regions, is a well-characterized epigenetic marker related to gene expression regulation in eukaryotes. Usually, methylation levels in the promoter are inversely correlated with the expression levels of the corresponding genes. However, new studies have evaluated the role of DNA methylation in the first exon, revealing that exon methylation facilitates the transcriptional process, and impacts directly in genes expression [32]. Thus, it is understood that other regions of the gene, spite of promoter region, might act in the expression control with different methylation patterns.

In this context, some issues on the molecular level should be considered. Firstly, methylation may not be the chief mechanism involved in the regulation of activity of that gene in blood. Secondly, the analyzed regions, although carefully chosen, may not be crucial for the regulation of gene expression [9]. Thus, the impact of the methylation levels on *ADRB3* gene and its relationship with lipid profile variables and oxidative stress might be better elucidated through further studies of gene expression.

In summary the beta-adrenergic receptor 3 encoded by the *ADRB3* gene is involved in the regulation of lipolysis and thermogenesis mainly in adipose tissue which provides free fatty acids for thermogenesis, indicating the participation of the *ADRB3* gene in the regulation of body weight in humans. In the present study we analysed blood tissue and it is important to mention that blood is metabolically active tissue, with an important role in the adverse inflammatory and vascular consequences of adiposity, and is widely used for clinical diagnostic purposes [5]. It has been suggested that polymorphisms in beta 3 adrenoreceptor signal transduction, binding, or regulatory mechanism may result in diminished lipolytic response [33]. In relation to association to *ADRB3* methylation profile and body weight the studies are still scarce and this relation needs to be further studied.

Some limitations of the present study are relevant. Regarding the absence of relationship with lipid profile, oxidative stress and food intake, probably did not occured due to the higher prevalence of eutrophic individuals, lower prevalence of obesity from the degree III, and the status of all variables studied situated around 90% in reference values in the total sample. And as the obeses group was composed mostly by individuals with mild and moderate obesity (grade I e II), there was no representative group of severe obesity (grade III and IV) (n = 5) in present study. In addition, a stratification by nutritional status according with gender was not performed, reflecting the methylation levels of the *ADRB3* gene in leukocytes based in a representative adult population of both genders.

## Conclusions

In conclusion, it was demonstrated a negative relationship between methylation levels of the *ADRB3* gene in individuals eutrophic adults with serum levels of folic acid, as well as with the independent gender of BMI, however, it was not observed relationship between lipid profile, oxidative stress and food intake in individuals distributed in the three categories of BMI.

Regarding the absence of relationship with methylation levels of the *ADRB3* gene in the categories of overweight, mild and moderate obesity, the answer probably lies in the insufficient amount of body fat to initiate inflammatory processes and oxidative stress with a direct impact on methylation levels, what differently is found most times in exacerbated levels in severe obesity.

## Additional file

**Additional file 1.** Additional tables.

## Abbreviations

5-FTHF: 5-formyltetrahydrofolate
5-MTHF: 5-methyltetrahydrofolate
II DISANDNT/JP: Second Cycle of Diagnosis and Intervention on the Diet, Nutrition and Most Prevalent Noncommunicable Diseases among the Population of João Pessoa, Paraiba
*ADRB3*: gene encoding beta-3 adrenergic receptor
AHA: American Heart Association
BMI: body mass index
DHFR: dihydrofolate reductase
HDL: high density lipoprotein
HRM: high resolution melting
LDL: low density lipoprotein
MDA: malondialdehyde
PCR: polymerase chain reaction
PPGCN: Post Graduate Program in Nutrition Sciences
R24h: 24-hour dietary recall
RBC: red blood cells
TAC: total antioxidant capacity
TC: total cholesterol
WC: waist circumference
WHO: World Health Organization
WHR: waist-to-hip ratio
WHtR: waist-to-height ratio
UFPB: Federal University of Paraíba
VAT: visceral adipose tissue
