# Supplementary material for: Decrease of the DNA methylation levels of the ADRB3 gene in leukocytes is related with serum folate in eutrophic adults
Source: J Transl Med. 2018 Jun 5;16:152. doi: 10.1186/s12967-018-1529-0 (PMC5987450; doi:10.1186/s12967-018-1529-0)
Supplement: Supplementary file 1 — Additional file 1. Additional tables. [file 12967_2018_1529_MOESM1_ESM.docx]

Additional Material

| **Table S1.** Multiple regression analysis of methylation levels of the *ADRB3* gene with lipid profile and oxidative stress of eutrophic adult individuals (n = 114). | | | | |
| --- | --- | --- | --- | --- |
| Methylation levels | | | | |
|  | Coefficient | CI 95% | Statistics t | p-Value |
| Gender | -0.15 | [-0.2047±-0.0953] | -2.79 | 0.0070* |
| Age | 0.00 | [-0.0021±0.0021] | 0.23 | 0.8214 |
| Total Cholesterol (mg/dl) | -0.00 | [-0.0009±0.0009] | -0.11 | 0.9120 |
| LDL (mg/dl) | -0.00 | [-0.0065±0.0065] | -0.48 | 0.6350 |
| HDL (mg/dl) | 0.00 | [-0.0026±0.0026] | 1.82 | 0.0734 |
| Triglycerides (mg/dl) | 0.00 | [-0.0005±0.0005] | 0.28 | 0.7777 |
| TAC (%) | 0.04 | [-0.1483±0.2283] | 0.22 | 0.8303 |
| MDA | -0.01 | [-0.0412±0.0212] | -0.42 | 0.6771 |
| Alpha-1 acid glycoprotein (mg/dl) | 0.00 | [-0.0012±0.0012] | 0.01 | 0.9958 |
| Homocysteine (micromol/l) | 0.00 | [-0.0018±0.0018] | 0.47 | 0.6392 |
| Serum folate (ng/ml) | -0.00 | [-0.0048±0.0048] | -1.49 | 0.1403 |
| Vitamin B12 (pg/ml) | -0.00 | [-0.0002±0.0002] | -0.21 | 0.8322 |

Values are expressed as the coefficient and CI (95%) and significance is at p < 0.05. The multiple regression analysis based on model 1 after adjustment for gender and age, observed that there was only relationship for methylation levels of *ADRB3* gene and male gender in the group of eutrophics adults.

| **Table S2.** Multiple regression analysis of methylation levels of the *ADRB3* gene with lipid profile and oxidative stress of adult individuals with overweight (n = 95). | | | | |
| --- | --- | --- | --- | --- |
| Methylation levels | | | | |
|  | Coefficient | CI 95% | Statistics t | p-Value |
| Gender | -0.07 | [-0.1283±-0.0117] | -1.19 | 0.2420 |
| Age | -0.00 | [-0.0024±0.0024] | -0.47 | 0.6420 |
| Total Cholesterol (mg/dl) | 0.00 | [-0.0010±0.0010] | 1.21 | 0.2340 |
| LDL (mg/dl) | -0.00 | [-0.0007±0.0007] | -1.15 | 0.2550 |
| HDL (mg/dl) | 0.00 | [-0.0029±0.0029] | 0.56 | 0.5800 |
| Triglycerides (mg/dl) | 0.00 | [-0.0004±0.0004] | 0.10 | 0.9180 |
| TAC (%) | 0.04 | [-0.1536±0.2336] | 0.22 | 0.8310 |
| MDA | -0.04 | [-0.0782±-0.0019] | -1.16 | 0.2540 |
| Alpha-1 acid glycoprotein (mg/dl) | -0.00 | [-0.0016±0.0016] | -0.57 | 0.5730 |
| Homocysteine (micromol/l) | -0.00 | [-0.0037±0.0037] | -0.91 | 0.3700 |
| Serum folate (ng/ml) | 0.00 | [-0.0064±0.0064] | 0.76 | 0.4500 |
| Vitamin B12 (pg/ml) | 0.00 | [-0.0003±0.0003] | 0.48 | 0.6350 |

Values are expressed as the coefficient and CI (95%). The multiple regression analysis based on model 1 after adjustment for gender and age, was used for to evaluate significant relationships remain between methylation levels of *ADRB3* gene and lipid profile and oxidative stress in the group of overweight adults.

| **Table S3.** Multiple regression analysis of methylation levels of the *ADRB3* gene with lipid profile and oxidative stress of obese adult individuals (n= 53). | | | | |
| --- | --- | --- | --- | --- |
| Methylation levels | | | | |
|  | Coefficient | CI 95% | Statistics t | p-Value |
| Gender | 0.05 | [-0.0342±0.1342] | 0.58 | 0.5675 |
| Age | 0.00 | [-0.0035±0.0035] | 0.37 | 0.7135 |
| Total Cholesterol (mg/dl) | -0.00 | [-0.0011±0.0011] | -0.77 | 0.4492 |
| LDL (mg/dl) | 0.00 | [-0.0009±0.0009] | 1.39 | 0.1777 |
| HDL (mg/dl) | -0.00 | [-0.0056±0.0056] | -0.55 | 0.5876 |
| Triglycerides (mg/dl) | -0.00 | [-0.0006±0.0006] | -0.37 | 0.7115 |
| TAC (%) | 0.17 | [-0.0797±0.4197] | 0.69 | 0.4993 |
| MDA | -0.03 | [-0.0898±0.0298] | -0.52 | 0.6065 |
| Alpha-1 acid glycoprotein (mg/dl) | -0.00 | [-0.0023±0.0023] | -0.11 | 0.9159 |
| Homocysteine (micromol/l) | -0.01 | [-0.0149±-0.0050] | -1.32 | 0.1985 |
| Serum folate (ng/ml) | -0.01 | [-0.0173±-0.0027] | -0.92 | 0.3677 |
| Vitamin B12 (pg/ml) | 0.00 | [-0.0004±0.0004] | 0.56 | 0.5808 |

Values are expressed as the coefficient and CI (95%). The multiple regression analysis based on model 1 after adjustment for gender and age, was used for to evaluate significant relationships remain between methylation levels of *ADRB3* gene and lipid profile and oxidative stress in the group of obese adults.

| **Table S4.** Multiple regression analysis of methylation levels of the *ADRB3* gene with variables of food intake of eutrophic adult individuals (n = 114). | | | | |
| --- | --- | --- | --- | --- |
| Methylation levels | | | | |
|  | Coefficient | CI 95% | Statistics t | p-Value |
| Calories (kcal) | 0.01 | [-0.0014±0.0191] | 0.86 | 0.3980 |
| Dietary folate (mcg) | -0.01 | [-0.0410±0.0225] | -0.29 | 0.7740 |
| Monounsaturated fat (g) | -0.47 | [-1.6880±0.7504] | -0.38 | 0.7050 |
| Oleic acid (g) | -0.54 | [-2.4909±1.4087] | -0.28 | 0.7840 |
| Omega 3 (g) | -4.81 | [-11.8135±2.1888] | -0.69 | 0.5000 |
| Omega 6 (g) | 3.00 | [1.1749±4.8296] | 1.64 | 0.1170 |
| Saturated fat (g) | -0.01 | [-0.7547±0.7357] | -0.01 | 0.9900 |
| Cholesterol (mg) | -0.08 | [-0.2323±0.0662] | -0.56 | 0.5850 |
| Trans fat (g) | 3.18 | [-5.7526±12.1124] | 0.36 | 0.7260 |

Values are expressed as the coefficient and CI (95%). The multiple regression analysis based on model 2 was used for to evaluate relationship between food intake and methylation levels of *ADRB3* gene in the group of eutrophics adults.

| **Table S5.** Multiple regression analysis of methylation levels of the *ADRB3* gene with variables of food intake of adult individuals with overweight (n = 95). | | | | |
| --- | --- | --- | --- | --- |
| Methylation levels | | | | |
|  | Coefficient | CI 95% | Statistics t | p-Value |
| Calories (kcal) | 0.00 | [-0.0033±0.0074] | 0.38 | 0.7048 |
| Dietary folate (mcg) | 0.00 | [-0.0278±0.0236] | -0.08 | 0.9349 |
| Monounsaturated fat (g) | 0.10 | [-0.5882±0.7917] | 0.15 | 0.8836 |
| Oleic acid (g) | 0.49 | [-0.3924±1.3809] | 0.56 | 0.5808 |
| Omega 3 (g) | -4.79 | [-12.3345±2.7628] | -0.63 | 0.5302 |
| Omega 6 (g) | 0.49 | [-0.2519±1.2397] | 0.66 | 0.5121 |
| Saturated fat (g) | -0.25 | [-0.6603±0.1621] | -0.61 | 0.5485 |
| Cholesterol (mg) | -0.18 | [-0.2785±-0.0871] | -1.91 | 0.0644 |
| Trans fat (g) | -1.94 | [-8.0941±4.2085] | -0.32 | 0.7540 |

Values are expressed as the coefficient and CI (95%). The multiple regression analysis based on model 2 was used for to evaluate relationship between food intake and methylation levels of *ADRB3* gene in the group of overweight adults.

| **Table S6.** Multiple regression analysis of methylation levels of the *ADRB3* gene with variables of food intake of obese adult individuals (n= 53). | | | | |
| --- | --- | --- | --- | --- |
| Methylation levels | | | | |
|  | Coefficient | CI 95% | Statistics t | p-Value |
| Total fat (g) | 0.00 | [-0.1987±0.2087] | 0.02 | 0.9808 |
| Calories (kcal) | 0.00 | [-0.0065±0.0056] | -0.07 | 0.9418 |
| Dietary folate (mcg) | -0.05 | [-0.0944±-0.0085] | -1.20 | 0.2466 |
| Vitamin B12 (mcg) | 0.88 | [-2.2099±3.9724] | 0.29 | 0.7788 |
| Monounsaturated fat (g) | 0.20 | [-0.7909±1.1951] | 0.20 | 0.8410 |
| Oleic acid (g) | -0.19 | [-1.1437±0.7726] | -0.19 | 0.8486 |
| Omega 3 (g) | 1.43 | [-4.5825±7.4493] | 0.24 | 0.8144 |
| Omega 6 (g) | -0.24 | [-1.0214±0.5469] | -0.30 | 0.7657 |
| Saturated fat (g) | 0.67 | [-0.0557±1.4028] | 0.92 | 0.3679 |
| Cholesterol (mg) | 0.04 | [-0.1034±0.1821] | 0.28 | 0.7859 |
| Trans fat (g) | -2.17 | [-7.2626±2.9210] | -0.43 | 0.6749 |

Values are expressed as the coefficient and CI (95%). The multiple regression analysis based on model 2 was used for to evaluate relationship between food intake and methylation levels of *ADRB3* gene in the group of obese adults.
